# Renal asymmetric dimethylarginine inhibits fibrosis

**DOI:** 10.1002/2211-5463.12949

**Published:** 2020-08-30

**Authors:** Ming Wu, Meijie Yuan, Yanzhe Wang, Bo Tan, Di Huang, Chen Wang, Yun Zou, Chaoyang Ye

**Affiliations:** ^1^ Department of Nephrology TCM Institute of Kidney Disease of Shanghai University of Traditional Chinese Medicine Key Laboratory of Liver and Kidney Diseases Ministry of Education Shanghai Key Laboratory of Traditional Chinese Clinical Medicine Shuguang Hospital Affiliated to Shanghai University of Traditional Chinese Medicine China; ^2^ Department of Nephrology The First Hospital of Hebei Medical University Shijiazhuang China; ^3^ College of Integrated Traditional Chinese and Western Medicine Hebei University of Chinese Medicine Shijiazhuang China; ^4^ Clinical Pharmacokinetic Laboratory Shuguang Hospital Affiliated to Shanghai University of Traditional Chinese Medicine China

**Keywords:** ADMA, CKD, DDAH‐1, DDAH‐2, renal fibrosis

## Abstract

Chronic kidney disease (CKD) is a worldwide public health problem that is caused by repeated injuries to the glomerulus or renal tubules. Renal fibrosis commonly accompanies CKD, and it is histologically characterized by excessive deposition of extracellular matrix proteins, such as fibronectin and collagen I, in interstitial areas. Indirect *in vivo* experimental data suggest that renal asymmetric dimethylarginine (ADMA) exerts antifibrotic activity in CKD. In this study, we aimed to demonstrate that renal ADMA has a direct effect on fibrosis *in vivo*. Normal saline, ADMA, nonsense control siRNA, Ddah1 siRNA or Ddah2 siRNA was administered into the kidney through the left ureter in a mouse model of unilateral ureteral obstruction (UUO). UUO kidneys were harvested at day 1 or 7. Western blotting was performed to assess the expression of ADMA, DDAH1 and DDAH2 and the expression of fibrotic markers, such as fibronectin, collagen I, α‐smooth muscle actin, phosphorylation of Smad3 and connective tissue growth factor. Masson’s trichrome staining was used to further evaluate renal fibrosis. We observed that intrarenal administration of ADMA increased the renal accumulation of ADMA and attenuated renal fibrosis at days 1 and 7. Knockdown of *Ddah1* or *Ddah2* increased the amount of ADMA in UUO kidneys and inhibited the expression of fibrotic proteins at days 1 and 7, which was further confirmed by Masson’s staining. Thus, our *in vivo* data suggest that renal ADMA exerts direct antifibrotic effects in a mouse model of UUO.

## Graphical abstract

Direct *in vivo* evidence regarding the effect of renal asymmetric dimethylarginine (ADMA) on fibrosis is lacking. We demonstrate here that intrarenal administration of ADMA attenuates renal fibrosis in unilateral ureteral obstruction mice. Knockdown of *Ddah1* or *Ddah2* in unilateral ureteral obstruction kidneys increased the amount of ADMA and inhibited the expression of fibrotic proteins. Thus, renal ADMA may exert antifibrotic effects in renal tubulointerstitial fibrosis.

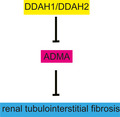

**Abbreviations**
ADMA: asymmetric dimethylarginine
Col‐I: collagen I
CTGF: connective tissue growth factor
DDAH: dimethylarginine dimethylaminohydroxylase
FN: fibronectin
NC: nonsense control
NS: normal saline
PRMT: type I protein arginine methyltransferase
α‐SMA: α‐smooth muscle actin
pSmad3: phosphorylation of Smad3
Smad3: decapentaplegic homolog 3
TGF‐β: transforming growth factor β
UUO: unilateral ureteral obstruction

Chronic kidney disease (CKD) is a worldwide public health problem that is caused by repeated injuries to glomerulus or renal tubules [1]. Renal fibrosis is the common pathway of all kinds of CKD, and it is histologically characterized by excessive deposition of extracellular matrix proteins, such as fibronectin (FN) and collagen I (Col‐I) in interstitial areas [2, 3]. Several animal models with different causes of CKD were used to study renal fibrosis. Hypertensive or diabetic animals were used to study renal fibrosis induced by glomerular injuries [4]. Unilateral ureteral obstruction (UUO) model is a classic animal model used to study tubulointerstitial fibrosis in the kidney [4]. It is related to human congenital obstructive kidney disease, which is the major cause of CKD in infants or children [4]. The cytokine transforming growth factor β (TGF‐β) plays a central role in the development of renal fibrosis by activating multiple downstream signaling pathways, for example, mothers against decapentaplegic homolog 3 (Smad3) and connective tissue growth factor (CTGF) pathways [5, 6]. In UUO kidneys, the expression of TGF‐β is upregulated in the proximal tubules, and transgenic overexpression of TGF‐β in the proximal tubules induced renal fibrosis [4].

Asymmetric dimethylarginine (ADMA) is an endogenous nitric oxide synthase inhibitor that is produced by type I protein arginine methyltransferases (PRMTs) and metabolized by dimethylarginine dimethylaminohydroxylase (DDAH), which consists of two isoforms, DDAH1 and DDAH2 [7, 8, 9]. Several *in vivo* and clinical studies have indicated that circulating ADMA is a risk factor and profibrotic in CKDs [10, 11]. However, our previous study showed that treatment with type I PRMT inhibitor reduced renal ADMA levels and promoted renal fibrosis, suggesting that renal ADMA is antifibrotic [12]. In agreement with this conclusion, Tomlinson *et al*. [8] showed that kidney‐specific knockout of *Ddah1* gene increased renal accumulation of ADMA and attenuated renal fibrosis in two different mouse models of CKD. Importantly, our *in vitro* study demonstrated the direct antifibrotic effect of ADMA on renal epithelial cells [12]. However, direct evidence supporting the antifibrotic effect of renal ADMA is still lacking.

In this study, we demonstrated the direct antifibrotic effect of renal ADMA on fibrosis *in vivo* using an intrarenal injection approach.

## Results

### Intrarenal administration of ADMA inhibited fibrosis in UUO mice

**Fig. 1:**
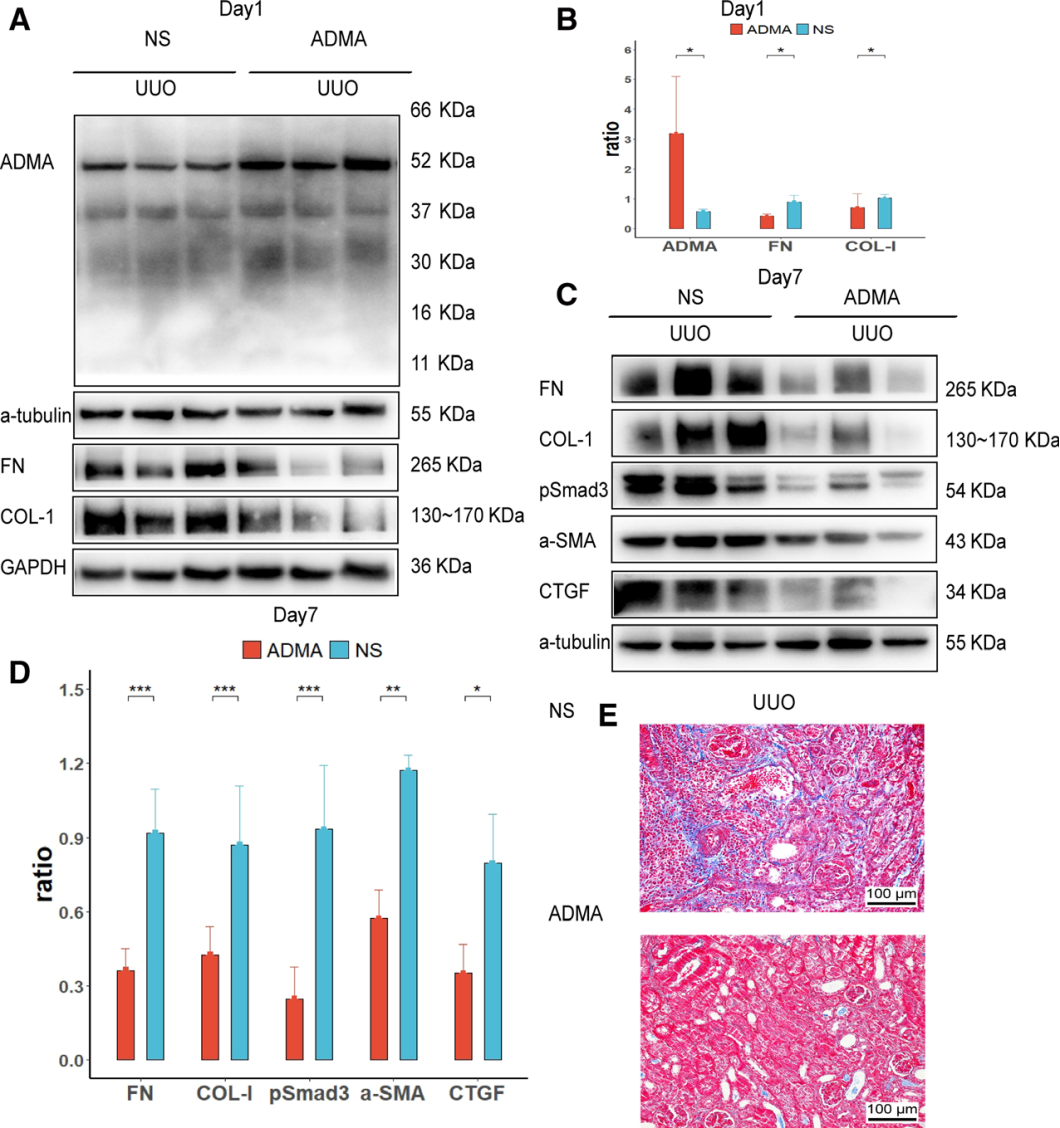
Intrarenal administration of ADMA inhibited fibrosis in UUO mice. Fifty microliters of NS (*n* = 11) or ADMA (0.6 mg, *n* = 11) was injected intrarenally to the left kidney, which was subjected to the UUO operation thereafter. Renal tissues were collected at day 1 (*n* = 4) or day 7 (*n* = 7) after UUO operation. The expressions of ADMA, FN and Col‐I at day 1 in the UUO kidney were measured by western blotting, and quantification of more than three blots is shown (A, B). The expressions of FN, Col‐I, CTGF, α‐SMA and pSmad3 at day 7 in the UUO kidney were measured by western blotting, and quantification of more than three blots is shown (C, D). Renal fibrosis was further evaluated by Masson’s trichrome staining at day 7 in the UUO kidney (E). One representative of three independent experiments is shown. Data represent mean ± SD. *P < 0.05 versus NS; **P < 0.01 versus NS; ***P < 0.001 versus NS. Scale bars: 100 μm. Statistical differences were analyzed by one‐way ANOVA or unpaired Student’s *t*‐test. A *P* value <0.05 was considered statistically significant.

Fifty microliters of ADMA (0.6 mg per mouse) or normal saline (NS) was delivered through the ureter to the left kidney, which was subjected to unilateral utero ligation (UUO) operation thereafter. At day 1, the total amount of ADMA was increased in UUO kidneys after ADMA administration, which was correlated with reduced expression of FN and Col‐I, two fibrotic markers (Fig. 1A,B).

At day 7, the expressions of FN, Col‐I, CTGF, α‐smooth muscle actin (α‐SMA) and phosphorylation of Smad3 (pSmad3) were reduced in ADMA‐treated UUO kidneys as compared with that in NS‐treated control kidneys as shown by western blotting (Fig. 1C,D). In line with these results, a mild interstitial fibrosis was detected in NS‐treated UUO kidneys by Masson’s trichrome staining, which was attenuated by ADMA at day 7 (Fig. 1E).

### Knockdown of *Ddah1* or *Ddah2* genes in the UUO kidney inhibited fibrosis

**Fig. 2:**
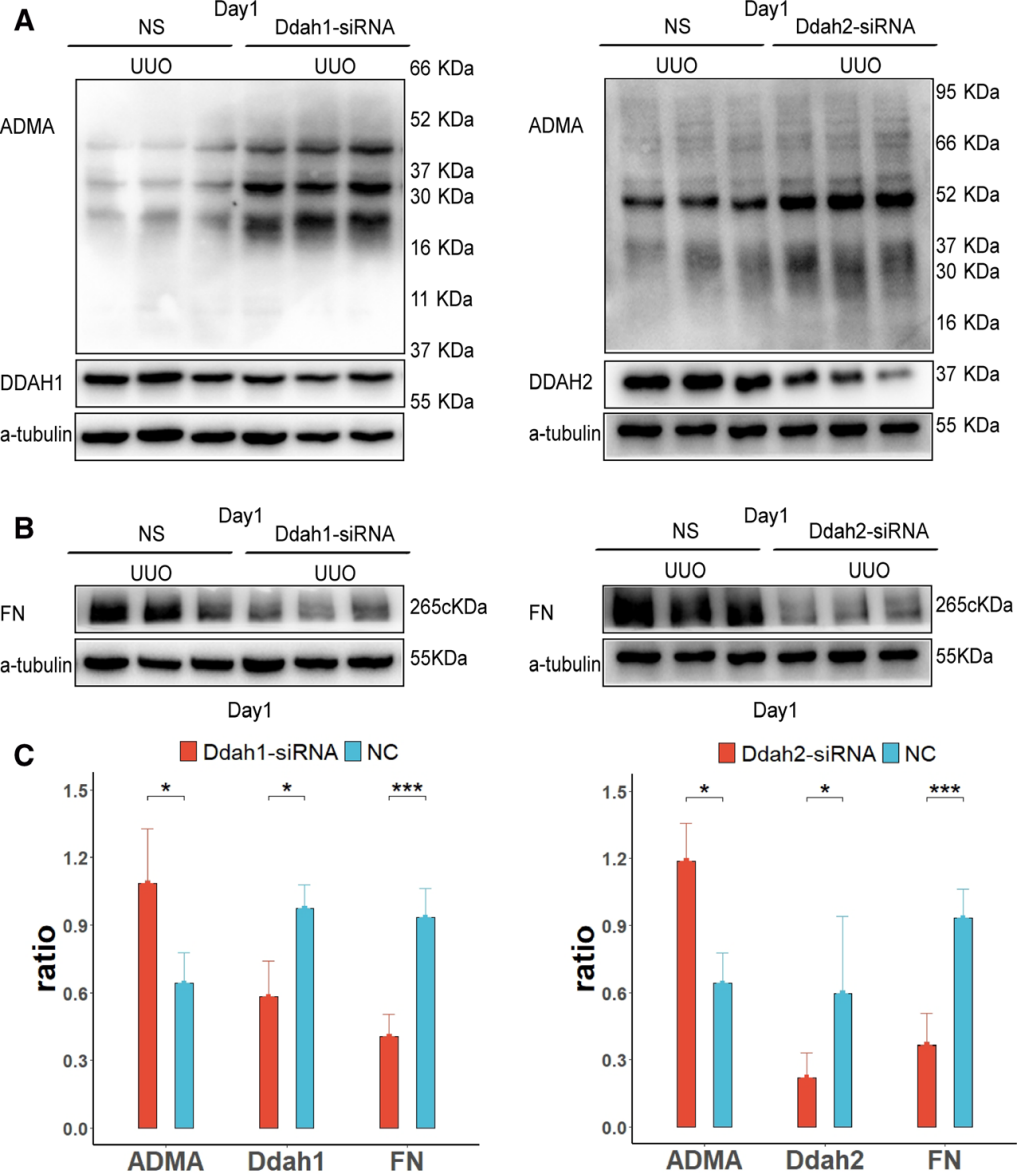
Knockdown of *Ddah1* or *Ddah2* gene inhibited renal fibrosis at day 1 after UUO operation. Fifty microliters of NC (*n* = 4), Ddah1 (*n* = 4) or Ddah2 (*n* = 4) siRNA was injected intrarenally to the left kidney, which was subjected to the UUO operation thereafter. Renal tissues were collected at day 1 after UUO operation. The expressions of ADMA, DDAH1, DDAH2 and FN at day 1 in the UUO kidney were measured by western blotting, and quantification of more than three blots is shown (A–C). One representative of three independent experiments is shown. Data represent mean ± SD. *P < 0.05 versus NC; ***P < 0.001 versus NC. Statistical differences were analyzed by one‐way ANOVA or unpaired Student’s *t*‐test. A *P* value <0.05 was considered statistically significant.

**Fig. 3:**
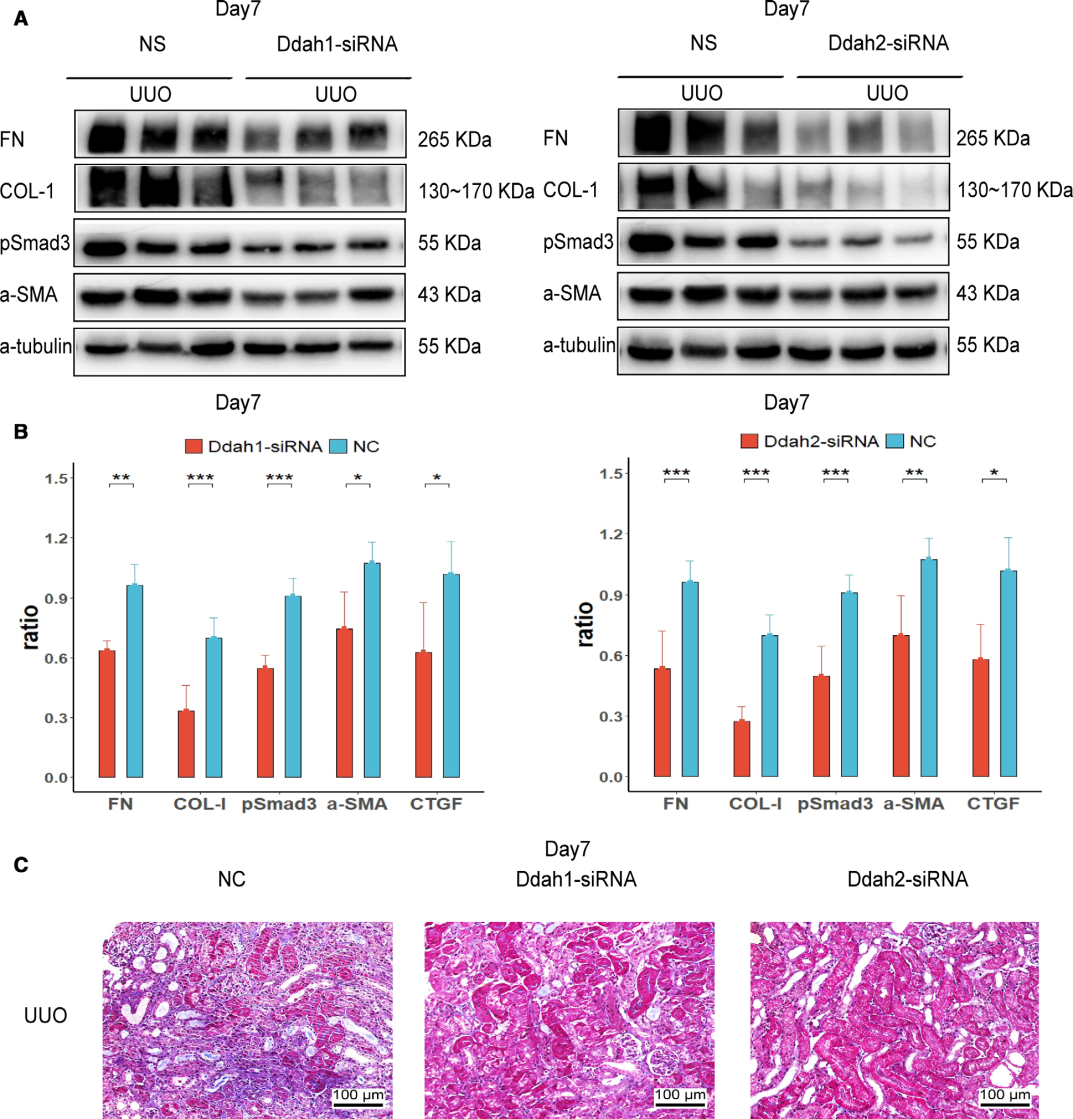
Knockdown of *Ddah1* or *Ddah2* gene inhibited renal fibrosis at day 7 after UUO operation. Fifty microliters of NC (*n* = 7), Ddah1 (*n* = 7) or Ddah2 (*n* = 7) siRNA was injected intrarenally to the left kidney, which was subjected to the UUO operation thereafter. Renal tissues were collected at day 7 after UUO operation. The expression of FN, Col‐I, CTGF, α‐SMA and pSmad3 at day 7 in the UUO kidney was measured by western blotting, and quantification of more than three blots is shown (A, B). Renal fibrosis was further evaluated by Masson’s trichrome staining at day 7 in the UUO kidney (C). One representative of three independent experiments is shown. Data represent mean ± SD. *P < 0.05 versus NC; **P < 0.01 versus NC; ***P < 0.001 versus NC. Scale bars: 100 μm. Statistical differences were analyzed by one‐way ANOVA or unpaired Student’s *t*‐test. A *P* value <0.05 was considered statistically significant.

To further determine the effect of renal ADMA on renal fibrosis, we injected nonsense control (NC), Ddah1 or Ddah2 siRNA to the UUO kidney through the left ureter. Down‐regulation of DDAH1 or DDAH2 protein was observed in Ddah1 or Ddah2 siRNA‐treated UUO kidneys, respectively, at day 1, which was correlated with increased production of ADMA in both groups as compared with that in the NC group (Fig. 2A,C). Treatment with Ddah1 or Ddah2 siRNA reduced the expression of FN at day 1 in UUO kidneys (Fig. 2B,C). At day 7, the expressions of FN, Col‐I and other fibrotic markers, CTGF, α‐SMA and pSmad3, were significantly reduced by Ddah1 or Ddah2 siRNA in UUO kidneys as compared with that in NC siRNA‐treated kidneys (Fig. 3A,B). Masson’s staining showed that positive signals in renal interstitial areas were attenuated by Ddah1 or Ddah2 siRNA as compared with that in the NC groups (Fig. 3C).

## Discussion

The effect of circulating and renal ADMA is opposite on renal fibrosis. The profibrotic effect of plasma ADMA was proved by direct administration of ADMA or injection of virus‐expressing DDAH1 in CKD animal models [10, 13]. The profibrotic mechanisms of circulating ADMA are probably related to the glomerular and vascular injuries induced in the nephrectomized CKD model [10, 13]. We and others demonstrated the antifibrotic effect of renal ADMA in two animal models of CKD by indirectly decreasing or increasing renal ADMA levels [8, 12]. The antifibrotic mechanism of renal ADMA is related to the reduced NO production in the proximal renal tubules, which in turn downregulates the expression of UMOD in the downstream thick ascending limb and thus exerts renal protection [8, 12]. The direct evidence supporting the inhibitory effect of ADMA on renal fibrosis was further provided by our *in vitro* studies [12]. We showed that ADMA inhibited fibrotic responses of HK2 human renal epithelial cells and NRK‐49F rat renal fibroblasts [12].

In this study, we showed that intrarenal injection of ADMA attenuated renal fibrosis at days 1 and 7 after the UUO operation as shown by western blotting and Masson’s staining. Moreover, kidney‐specific knockdown of *Ddah1* or *Ddah2* gene increased the amount of renal ADMA in UUO mice, which was correlated with the reduced expression of fibrotic markers or weakened Masson’s staining at days 1 and 7 in UUO kidneys. Thus, we conclude that renal ADMA is antifibrotic, which is in line with the conclusion provided by previous *in vitro* studies or indirect evidence using type I PRMT inhibitors or kidney‐specific deletion of *Ddah1* gene in mouse models of renal fibrosis [8, 12]. Interestingly, a recent study showed that reduction of renal ADMA levels by intrarenal injection of adenovirus‐expressing DDAH‐1 ameliorates diabetic nephropathy [14]. Diabetic nephropathy is mostly caused by the injury in the glomerulus, while we and others studied the effect of ADMA on renal interstitial fibrosis and focused on renal tubular‐related injuries. Thus, the antifibrotic effect of renal ADMA could be limited to the certain pathology of CKD.

To control the side effect of ADMA for treating renal fibrosis in patients, the primary cause of CKD should be understood, and a better strategy to target renal epithelial cells or fibroblasts in the kidney should be designed. Gene therapy targeting specific cells in the kidney using adenoviral promoter fragments was successfully tested in animals [15]. However, the clinical application of gene therapy against renal fibrosis has not been developed to date. In the current stage, the most practical strategy using our results is to avoid the reduction of ADMA in renal tubulointerstitial areas that might be caused by pharmaceutical agents.

## Materials and Methods

### Animals and UUO operation

Male C57 mice (Specific‐pathogen‐free grade, 20–25 g) were purchased from Shanghai SLAC Laboratory Animal Co., Ltd., Shanghai,China. Animals were kept in the animal center of Shanghai University of Traditional Chinese Medicine according to local regulations and guidelines.

After anesthesia with sodium pentobarbital (8 mg·kg^−1^ intraperitoneally), the left mouse kidney was exposed by an incision. UUO operation was performed through twice ligation of the left ureter with 4‐0 nylon sutures. Animal experiments described herein were endorsed by the animal experimentation ethics committee of Shanghai University of Traditional Chinese Medicine (PZSHUTCM18111601).

Mice were randomly divided into five groups: (a) UUO + NS (*n* = 11), (b) UUO + ADMA (*n* = 11), (c) UUO + NC siRNA (*n* = 11), (d) UUO + Ddah1 siRNA (*n* = 11), and (e) UUO + Ddah2 siRNA (*n* = 11) group. Four mice from each group were sacrificed at day 1, and the rest of mice were sacrificed at day 7 for tissue collection.

### *In vivo* drug and siRNA administration

ADMA (C5216) was purchased from APExBIO (Houston, TX, USA) and dissolved in NS. NC siRNA, mouse DDAH1 siRNA (TGGCCGA TTCTTTGCATTTAA) and mouse DDAH2 siRNA (GGCAGUGUCUCGAGAACUU) were synthesized by Hanbio Biotechnology (Shanghai, China). For siRNA transfection, *in vivo*‐jetPEI reagent (Polyplus‐transfection Inc., New York, NY, USA) complex (0.16 µL of *in vivo*‐jetPEI per µg nucleic acid) was prepared according to the manufacturer’s instructions. For monitoring the injection process, 0.04% trypan blue dye (A601140; Sangon, Shanghai, China) was added into the ADMA or siRNA complex. Fifty microliters of ADMA (12 mg·mL^−1^) or siRNA solution (0.1 mg·mL^−1^) was injected retrogradely once into the left kidney via the ureter. UUO was performed right after the injection.

### Masson’s trichrome

Mouse kidneys were fixed in 4% paraformaldehyde and further embedded in paraffin. Masson’s trichrome staining was performed using a standard protocol. In brief, the 4‐μm‐thick sections of paraffin‐embedded kidney tissue were stained with hematoxylin and then with ponceau red liquid dye acid complex, which was followed by incubation with phosphomolybdic acid solution. Finally, the tissue was stained with aniline blue liquid and acetic acid. Images were obtained with the use of a microscope (Nikon 80i; Tokyo, Japan).

### Western blotting analysis

Renal protein was extracted from the medulla and cortex of mouse kidneys. The protein concentration was measured by the Bradford method, and the supernatant was dissolved in 5× SDS/PAGE loading buffer (P0015L; Beyotime Biotech, Nantong, China). Samples were subjected to SDS/PAGE gels. After electrophoresis, proteins were electrotransferred to a poly(vinylidene difluoride) membrane (Merck Millipore, Darmstadt, Germany), which was incubated in the blocking buffer (5% nonfat milk, 20 mm Tris–HCl, 150 mm NaCl, pH 8.0, 0.01% Tween 20) for 1 h at room temperature and was followed by incubation with anti‐FN (1 : 1000, ab23750; Abcam, Cambridge, MA, USA), anti‐CTGF (1 : 1000, sc‐373936; Santa Cruz Biotechnology, Santa Cruz, CA, USA), anti‐Col‐I (1 : 500, AF7001, sc‐293182; Santa Cruz), anti‐α‐SMA (1 : 1000, ET1607‐53; HUABIO, Hangzhou, China), anti‐GAPDH (1 : 5000, 60004‐1‐lg; Proteintech, Wuhan, China), anti‐ADMA (1 : 1000, 13522; Cell Signaling Technology (CST), Boston, MA, USA) or anti‐α‐tubulin (1 : 1000, AF0001; Beyotime) overnight at 4℃. Binding of the primary antibody was detected by an enhanced chemiluminescence method (BeyoECL Star, P0018A; Beyotime) using horseradish peroxidase‐conjugated secondary antibodies [goat anti‐rabbit IgG, 1 : 1000, A0208 (Beyotime) or goat anti‐mouse IgG, 1 : 1000, A0216 (Beyotime)]. The quantification of protein expression was performed using Quantity One Analyzer (Bio‐Rad, Laboratories, Hercules, CA, USA).

### Statistical analysis

Results were presented as mean ± SD. Differences among multiple groups were analyzed by one‐way ANOVA, and comparison between two groups was performed by unpaired Student’s *t*‐test, using statistical software spss 18.0 (SPSS Inc., Chicago, IL, USA). A *P*‐value <0.05 was considered statistically significant.

## Conflict of interest

The authors declare no conflict of interest.

## Author contributions

MW and CY conceived and coordinated the study. MW and CY wrote the paper. MW, MY, YW, BT, DH, YZ, and CW designed, performed and analyzed the animal experiments. MY performed and analyzed the western blotting. All authors reviewed the results and approved the final version of the manuscript.

## Data Availability

Data are available from the corresponding author upon request.
